# Retained Needle in the AirSeal Trocar During Robot-Assisted Laparoscopic Radical Prostatectomy: Lessons Learned

**DOI:** 10.1089/cren.2018.0034

**Published:** 2018-07-01

**Authors:** Matthew Moynihan, Alireza Moinzadeh

**Affiliations:** Lahey Hospital & Medical Center, Burlington, Massachusetts.

**Keywords:** needle, trocar, prostatectomy, robotic, AirSeal

## Abstract

***Background:*** Unique case of retained needle in the AirSeal trocar during robot-assisted laparoscopic radical prostatectomy.

***Case Presentation:*** A 68-year-old male with prostate cancer underwent robot-assisted laparoscopic radical prostatectomy. Upon laparoscopic removal of final intra-abdominal suture by bedside assistant, needle became dislodged from suture and was unable to be located after a standard systematic search. Ultimately, needle was found caught in the assistant's AirSeal trocar device.

***Conclusion:*** Intraoperative loss of a foreign body should include inspection, and possible radiographic evaluation, of the trocar mechanism as part of a complete systematic approach.

## Introduction and Background

Radical prostatectomy remains a viable option for management of intermediate- and high-risk localized prostate cancer. The robot-assisted laparoscopic approach is now considered a standard of care option for surgical care of this disease entity, as it is associated with low morbidity and swift recovery.^1^ In performing the procedure, our institution frequently uses the SurgiQuest AirSeal trocar device as an assistant port. We present a case of a retained surgical needle in this assistant trocar port and offer recommendations for intraoperative evaluation and identification of a missing needle during laparoscopic or robotic surgical procedures.

## Presentation of Case

A 68-year-old male with a medical history of hypertension, dyslipidemia, glaucoma, lumbar radiculopathy, and nephrolithiasis status postureteroscopy who was referred to the urology service for an abnormal digital rectal examination (DRE) and elevated prostate specific antigen (PSA) found on routine screening by his primary care physician. At time of referral his PSA was 4.0 with a PSA density of 0.13. Less than 2 years prior his PSA was found to be 2.9 on routine screening. At baseline, he was determined to have moderately symptomatic urinary symptoms as determined by the International Prostate Symptom Score (value = 12) and reported mild erectile dysfunction according to the International Index of Erectile Function (value = 20). He denied any family history of prostate cancer.

Pertinent physical examination findings were notable for a BMI of 24.8 and a palpable right-sided prostatic nodule on DRE. Preoperative laboratory evaluation was all within normal limits.

He was evaluated by the urology service and was recommended to undergo a transrectal ultrasound guided prostatic biopsy. Ultimately, his biopsy demonstrated 10/12 core biopsies positive for Gleason 3 + 4 = 7 prostatic adenocarcinoma, with the core from the left lateral apex showing Gleason 4 + 3 = 7. His Eastern Cooperative Oncology Group (ECOG) performance status was determined to be Grade 0. His clinical stage according to the American Joint Committee on Cancer at this time was determined to be T2a. Given his biopsy findings suggesting high-volume prostatic adenocarcinoma of intermediate risk, he underwent prostatic magnetic resonance imaging that demonstrated no discrete lesions.

After careful consideration of all options, the patient elected to pursue a minimally invasive surgical intervention and underwent robot-assisted laparoscopic prostatectomy with bilateral pelvic lymph node dissection. This was performed utilizing the *da Vinci Xi* Surgical System (Intuitive Surgical, Sunnyvale, CA). Intraperitoneal access was obtained through an open Hasson technique. Four robotic trocars were subsequently placed under laparoscopic direct vision for the robot. A 5 mm assistant trocar was placed in the left-upper quadrant, and a 12 mm AirSeal port (SurgiQuest, ConMed Corporation, Milford, CT) was placed in the left lateral aspect of the patient's abdomen.

The key procedural aspects of the case were largely unremarkable. In brief, the prostate was approached posteriorly, first dissecting out the seminal vesicles and vas deferens. A bilateral limited pelvic lymph node dissection was performed. A partial nerve-sparing technique was accomplished bilaterally. The vesicourethral anastomosis was created using 3-0 Covidien V-Loc (Medtronic, New Haven, CT) barbed unidirectional suture. The anastomosis was tested *in situ* and appeared to be intact.

**FIG. 1.**
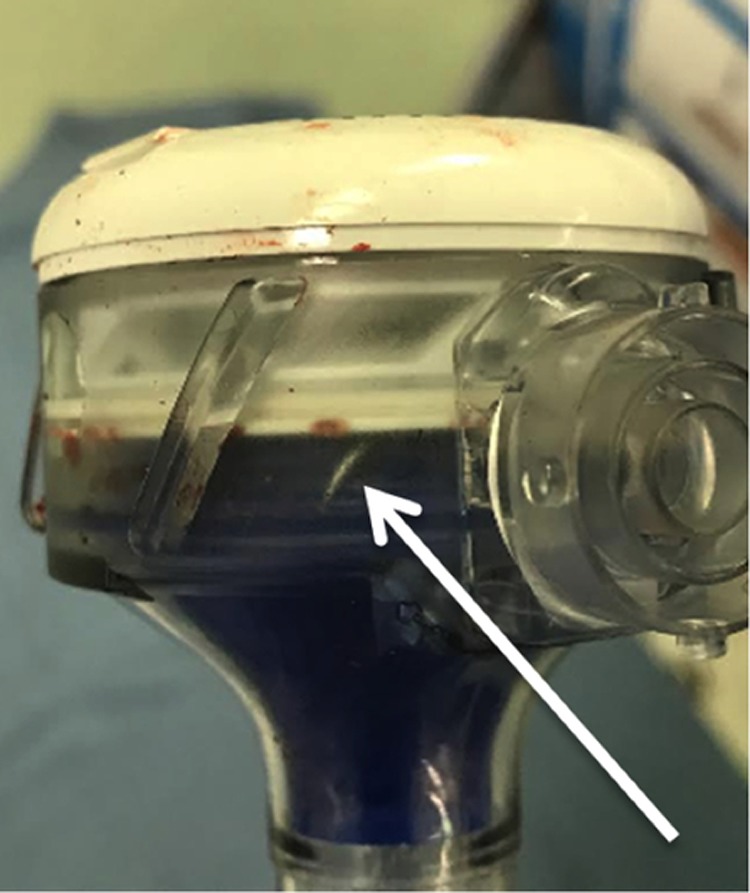
Picture demonstrating needle in trocar head (*white arrow*).

**FIG. 2.**
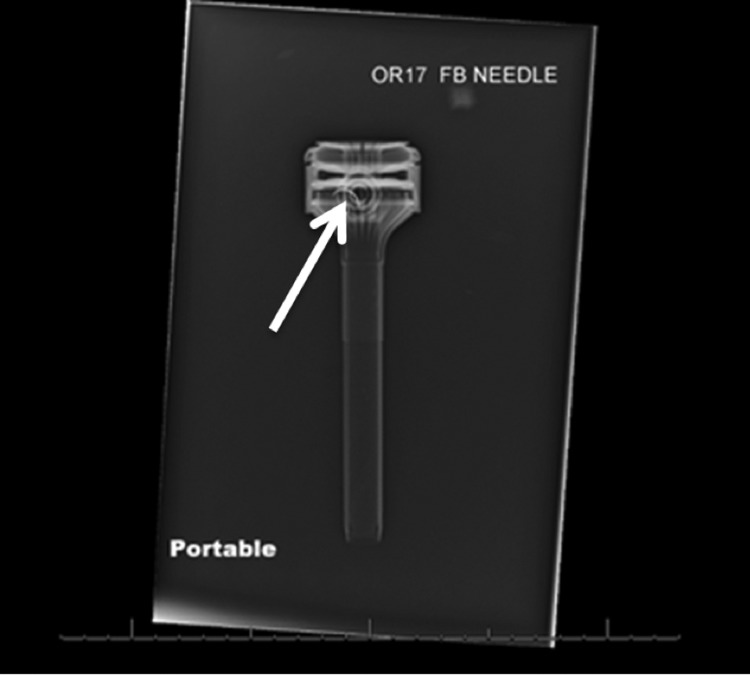
Portable radiograph showing needle in trocar head (*white arrow*).

At the conclusion of the case before undocking the *da Vinci* system, bilateral anti-lymphocele sutures were placed robotically using 3-0 Vicryl suture on an RB1 needle^2^ (Ethicon, Johnson & Johnson; Somerville, NJ). We typically remove any remnant suture by having the assistant grasp the suture with a laparoscopic needle driver ∼4 cm from the needle. During extraction of the final Vicryl suture with RB1 needle with a laparoscopic needle driver through the AirSeal trocar, resistance was met. Upon extraction of the suture by the assistant, no needle was noted on the suture of the 3-0 Vicryl. The robotic zero degree camera/lens was introduced into the AirSeal trocar to evaluate for a potential needle within the trocar. The needle was not visualized within the cannula. Careful laparoscopic examination of the abdomen in a systematic manner did not reveal the needle intra-abdominally. The RB1 needle was ultimately identified within semitransparent plastic of the inner aspect of the AirSeal trocar, separate from the working lumen (Fig. 1). Its location was confirmed visually and radiographically (Fig. 2). The trocar was disassembled with difficulty to clearly reveal the needle. The fascia was subsequently closed. At the conclusion of the case, all needle, instrument, and sponge counts were correct.

The patient returned to the office 11 days postoperatively for a follow-up visit. He underwent a cystogram, which demonstrated no extravasation from the vesicourethral anastomosis, and his indwelling urinary catheter was subsequently removed. At that time, he endorsed tolerating a regular diet, having regular bowel movements, ambulating without difficulty, and was avoiding narcotic pain medications. He denied any calf pain or swelling.

## Discussion and Literature Review

**FIG. 3.**
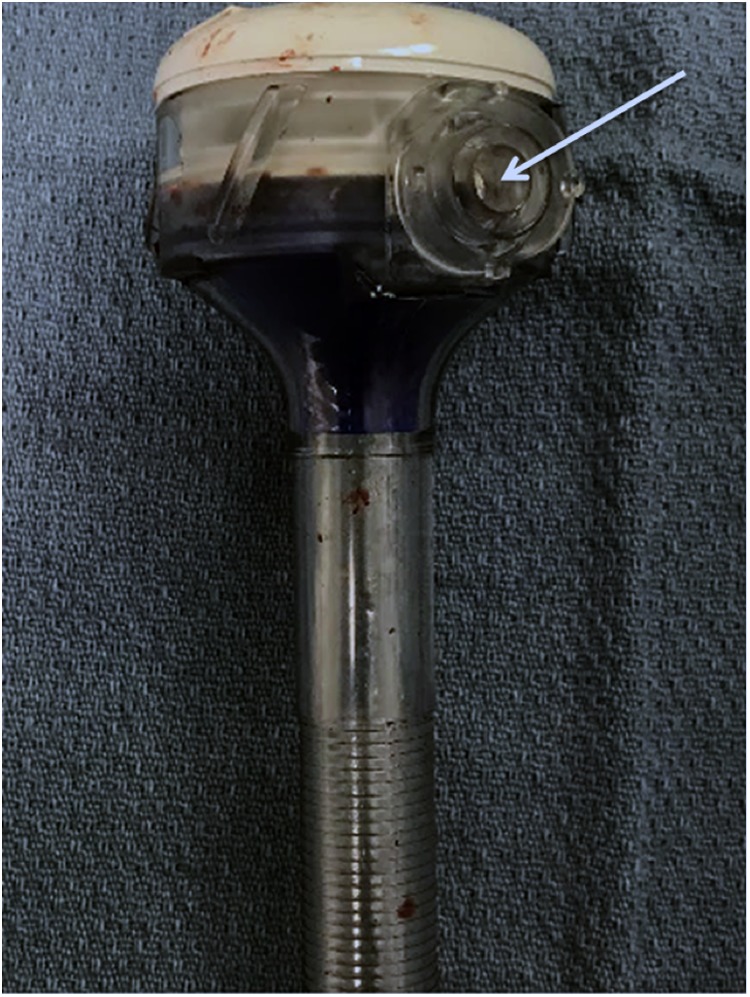
AirSeal trocar, with triple lumen connection side port visible (*white arrow*).

Use of the AirSeal system provides several advantages during minimally invasive surgery. The system allows for a stable pneumoperitoneum, constantly evacuates surgical smoke, and maintains high flow insufflation. This is accomplished utilizing a triple lumen tube set that attaches directly to the AirSeal port (Fig. 3). In the presented case, we believe that the constant air circulation and port design proved to have a limitation in allowing for entrapment of the needle. Once the needle was entrapped in the AirSeal trocar, the needle and suture separated.

Zaman and coworkers have recently noted that minimally invasive surgery using robotic systems, often with multiple bedside members and a large array of instruments, increases the chance of losing foreign bodies.^3^ A missing foreign body is an unnerving event which prompts an active and exhaustive search, ideally performed in a systematic manner to increase the chances of identification and retrieval. Jayadevan and associates recently suggested a lost needle identification protocol that includes initial operative field survey, radiograph for needle >13 mm, port inspection, anatomically systematic visual search, and floor/table sweep.^4^ However, there is no literature on the best manner in which to inspect a trocar. In this case, the needle was entrapped in an unexpected location within the inflow mechanism of the AirSeal port which was not visualized by laparoscopic inspection of the valveless AirSeal trocar. In fact, this is actually the second time a needle has become ensnared in the inflow mechanism of the AirSeal trocar over a 1 year period. To the best of our knowledge, the potential for needle loss within the AirSeal mechanism has never been reported. As such, it was not part of our checklist during a search for a missing needle.

We advocate that the trocar mechanism should be included in the systematic search for the missing needle. This may require both visual inspection of the round trocar head, evaluation of the trocar with a zero degree lens, and circumferential evaluation of the trocar head with a 30 degree lens. Finally, consideration should be given to radiographic imaging of the trocar. It is important to note that retained foreign bodies after abdominal operations are estimated to affect 1 per 1000 to 18,000 procedures^4^ and may lead to postoperative complications such as infection, hemorrhage, and pain in certain cases.

## Conclusion

Our experience with a lost needle in the AirSeal trocar calls attention to the need for evaluating the trocar device in laparoscopic surgery in the event of a missing foreign body. We advocate for inspection and possible radiographic evaluation of the trocar mechanism as a component of a complete systematic approach to locating a missing needle or foreign body in laparoscopic and robotic surgery.

## Abbreviations Used

DRE: digital rectal examination
PSA: prostate specific antigen
